# Suspected cutaneous adverse drug reactions reported with traditional medicines: analysis of data for United Nations Asia region from WHO VigiBase

**DOI:** 10.3389/fphar.2023.1088841

**Published:** 2023-05-30

**Authors:** Manish J. Barvaliya, A. C. Chetan, N. Chandan, Suman Kumar Ray, Harsha V. Hegde, Banappa S. Unger, Tejas K. Patel, Subarna Roy

**Affiliations:** ^1^ ICMR-National Institute of Traditional Medicine, Belagavi, Karnataka, India; ^2^ Department of Pharmacology, All India Institute of Medical Sciences, Gorakhpur, Uttar Pradesh, India

**Keywords:** traditional medicines, vigibase, pharmacovigilance, Stevens-Johnson syndrome, cutaneous adverse drug reactions, signal, spontaneous reporting

## Abstract

**Background:** Data on traditional medicine-induced cutaneous adverse drug reactions (ADRs) is very scarce. The current secondary analysis based on the WHO database (VigiBase) of individual case safety reports (ICSRs) focuses on the suspected cutaneous ADRs linked to traditional medicines (TMs).

**Methods:** All the ICSRs reported between 1st January 2016 and 30th June 2021 from the UN Asia region in VigiBase where at least one TM was suspected to cause cutaneous ADRs were included in the study. Data regarding demographic details, suspected drug, adverse reaction as per MedDRA term, the seriousness of the reaction, de-challenge, re-challenge, and clinical outcome for suspected cutaneous ADRs associated with TM were obtained from VigiBase and analyzed for frequency of reported events and suspected medicines.

**Findings:** Total 3,523 ICSRs with 5,761 ADRs related to “skin and subcutaneous tissue disorders” were included in the analysis. Amongst these, 6.8% of ICSRs were reported as serious. Pruritus (29.6%), rash (20.3%), urticaria (18.9%), and hyperhidrosis (3.3%) were commonly reported ADRs. *Artemisia argyi* H.Lév. and Vaniot. (14.9%), *Ginkgo biloba* L. (5.1%), *Vitis vinifera* L. (4%), *Vitex agnus-castus* L. (3.8%), *Silybum marianum* (L.), Gaertn (3.5%), and *Viscus album* L. (2.7%) were some commonly suspected TMs for cutaneous ADRs. There were 46 cases of Stevens-Johnson syndrome and toxic epidermal necrolysis reported with TMs during the study period. Death was reported in 5 ICSRs.

**Interpretation:** TMs are linked with various cutaneous ADRS ranging from pruritus to toxic epidermal necrolysis which may have serious consequences. TMs listed as suspected offending agents in this analysis, should be kept in mind while dealing with suspected cutaneous ADRs. Clinicians should be more vigilant in detecting and reporting events associated with TMs.

## Introduction

The use of plant, animal, and mineral-based materials as medicines based on local cultural knowledge and belief for diagnosis, treatment, and prevention of illnesses is called a traditional healthcare system and medicines of this system are called traditional medicines (TMs) (Fokunang, et al., 2011). There is a general belief that TMs are natural, safe and devoid of adverse effects as they are mainly botanicals and this leads to frequent indulging in self-medication either as stand-alone and/or with other therapies (Vickers et al., 2006). However, many adverse reactions associated with botanical drugs have been reported (Posadzki et al., 2013; Kalaiselvan et al., 2015; Fatima and Nayeem, 2016; Onder and Liperoti, 2016). Several TMs have been linked to the development of adverse drug reactions (ADRs) because they contain bioactive compounds which are pharmacologically active (de Boer et al., 2015; Venhuis et al., 2016).

According to the World Health Organization (WHO, 2023), pharmacovigilance (PV) is the science and practices of detecting, assessing, understanding, and preventing adverse effects or any other drug-related concern. For conventional pharmaceuticals, PV systems are well-established and integrated into healthcare and regulatory processes; however, this is not true for TMs (Barnes, 2003). However, the involvement of TMs alone or its combinations with conventional pharmaceuticals in suspected ADRs are made possible by spontaneous ADR reporting and active surveillance, through pharmacovigilance centers (Skalli and Soulaymani, 2012).

The skin is more commonly involved system in suspected ADRs. The incidence of suspected cutaneous ADRs is 1%–3% in developed countries and 2%–5% in developing countries (Modi et al., 2019). Although, cutaneous ADRs are frequently mild and benign, their early diagnosis with withdrawal of the causative drug at the earliest is crucial for avoiding a more serious problem. Angioedema, erythema multiforme, Stevens-Johnson syndrome (SJS), skin rashes, urticaria, itching, fixed drug eruption, and Toxic Epidermal Necrolysis (TEN) are some of the important cutaneous ADRs (Sharma et al., 2001; Barvaliya et al., 2011). SJS and TEN are uncommon but are severe forms of cutaneous ADRs that negatively impact the patient’s quality of life (Sharma et al., 2001). Various cutaneous reactions like TEN and dermatitis have been reported with traditional Chinese medicines (Lim and Thirumoorthy, 2005; Sen et al., 2010; Wu and Deng, 2013). In a prospective study conducted by Niang et al. (2015) in Africa, the authors, identified medicinal plant-related worsening of existing dermatitis, recurrence, and new onset dermatitis (Niang et al., 2015). Even though cutaneous ADRs are frequent, very little is known about cutaneous reactions associated with TMs. Detailed information about their frequency, severity, and long-term impact on health is lacking because of the high number of unreported cases. Retrospective epidemiology data are therefore needed to determine the medications linked to the risk of suspected cutaneous ADRs. VigiBase is a WHO global database of documented suspected ADRs in the form of individual case safety reports (ICSRs) (https://who-umc.org/vigibase/). Member countries contribute to VigiBase data by collecting suspected ADR reports through their PV programme. Hence, data from VigiBase can be useful to evaluate the pattern of reported cutaneous suspected adverse reactions linked with TMs. The present study analyzed the data of suspected cutaneous ADRs associated with various TMs reported in VigiBase by the United Nations Asia region.

## Materials and methods

The current study analyzed the de-identified secondary data from WHO VigiBase for suspected cutaneous ADRs due to TMs. Ethics review was exempted for the study. The authors obtained data from VigiBase through an agreement and the completion of required formalities with UMC, Sweden.

### Data source

Data of suspected cutaneous ADRs linked with TMs were obtained from VigiBase, which is the WHO global database of reported potential side effects of medicinal products, developed and maintained by Uppsala Monitoring Centre (UMC, 2023), Sweden (https://who-umc.org/media/yzpnzmdv/umc_caveat.pdf).

The scientific names of the botanical drugs were confirmed through the Medicinal Plants Names services by Kew Science (https://mpns.science.kew.org/mpns-portal/).

### Data set

All ICSRs reported between 1st January 2016 and 30th June 2021 from the UN Asia region with listed TMs [ATC code “V90: Unspecified Herbal and Traditional Medicine” and under the product type “herbal remedy-compositions of substances of natural origin (002)”] as suspected drugs were assessed. All ICSRs where at least one TM was suspected of causing the cutaneous ADRs were considered. ICSRs with missing notifier information were excluded from the analysis.

ICSRs were provided with information on demographic details (age, gender); Drug (WHO preferred trade name, base name, salt name; basis, dose, frequency, route, indication), ADR data [event type, event as per MedDRA terminologies time of onset, seriousness, de-challenge, re-challenge, outcome] and notifier.

### Key-terms

• TMs: plant and animal-based products in the natural form, either standalone or in combination, were considered TMs.
• Cutaneous ADRs: as per Medical Dictionary for Regulatory Activities (MedDRA, 2023), ICSRs in which the “skin and subcutaneous tissue disorders” was mentioned as system organ class (SOC) were considered as cutaneous ADRs. MedDRA Preferred Term (PT) was considered to describe various reactions.
• Causative drugs: cutaneous ADRs where at least one TM was mentioned as a “suspected” drug were considered. If TMs were mentioned as “concomitant” and “interacting” drugs in ICSRs, such ICSRs were not considered.
• Serious ADRs: all suspected ADRs noted as serious in the dataset were considered serious ADRs. ADRs leading to hospitalization/prolongation of hospitalization, life-threatening situations, disability, congenital malformation, and death of the patient were the various criteria for considering ADRs as serious; however, in the dataset, ADRs mentioned as serious without specifying the criteria for seriousness were considered.

### Data uniformity

The event term in the dataset was mentioned as per the MedDRA, which classifies the terms from System organ class (SOC) to Lowest Level Terms (LLT), which directly permits the assignment of MedDRA terms within a user database based on observation reported in practice. A symptom, sign, illness diagnosis, therapeutic indication, inquiry, surgical or medical treatment, and medical social or family history characteristic are each denoted by a different descriptor (single medical concept) at the “Preferred Terms” (PTs) level. The related PTs as a group forms High-Level Term (HLT), which is again clubbed into High-Level Group Term (HLGT). Finally, HLGTs are grouped into a common category called System Organ Class (SOC) (https://www.meddra.org/how-to-use/basics/hierarchy). The VigiMatch was used to identify duplicate information in a dataset based on matched and mismatched information between the pairs of reports through a statistical model (https://who-umc.org/research/vigimethods/). Moreover, VigiBase uses various medical and drug classifications like WHO-Drug, MedDRA, WHO ICD, and WHO-ART for ensuring uniform structural data entry from various member countries. Thus, the data quality during data retrieval for effective and accurate analysis is guaranteed (https://who-umc.org/research/vigimethods/).

### Data analysis

The data set was reviewed by two authors independently for accuracy and duplicate re-check. Identified duplicate reports were provided with the same report id after VigiMatch was run by VigiBase. In duplicate reports, all information matched except the notifier. We consider ADR reports from healthcare professionals in case of same report notified by different reporters. Data were presented in number and frequency for demographic data, commonly reported suspected cutaneous ADRs, TMs suspected to be causative, serious ADRs, TMs suspected of causing SJS/TEN, and clinical outcomes of ICSRs. In a large dataset, common (>1%) ADRs and medicines were presented in the analysis. Data were analyzed using the IBM SPSS software version 25.0.

## Results

A total of 28,507 ICSRs were retrieved with TMs as suspected causative drugs with at least one suspected ADR from VigiBase for United Nations Asia region. Amongst these, 5,686 ICSRs were related to Skin and subcutaneous tissue disorders in the MedDRA SOC, accounting for 19.9% of all reports. There were 8,588 reactions reported in 5,686 ICSRs pertaining to the skin. Reporter information was missing in 2,163 ICSRs which were excluded from the analysis. Thus, 3,523 ICSRs with 5,761 suspected cutaneous ADRs were considered for final analysis.

**FIGURE 1:**
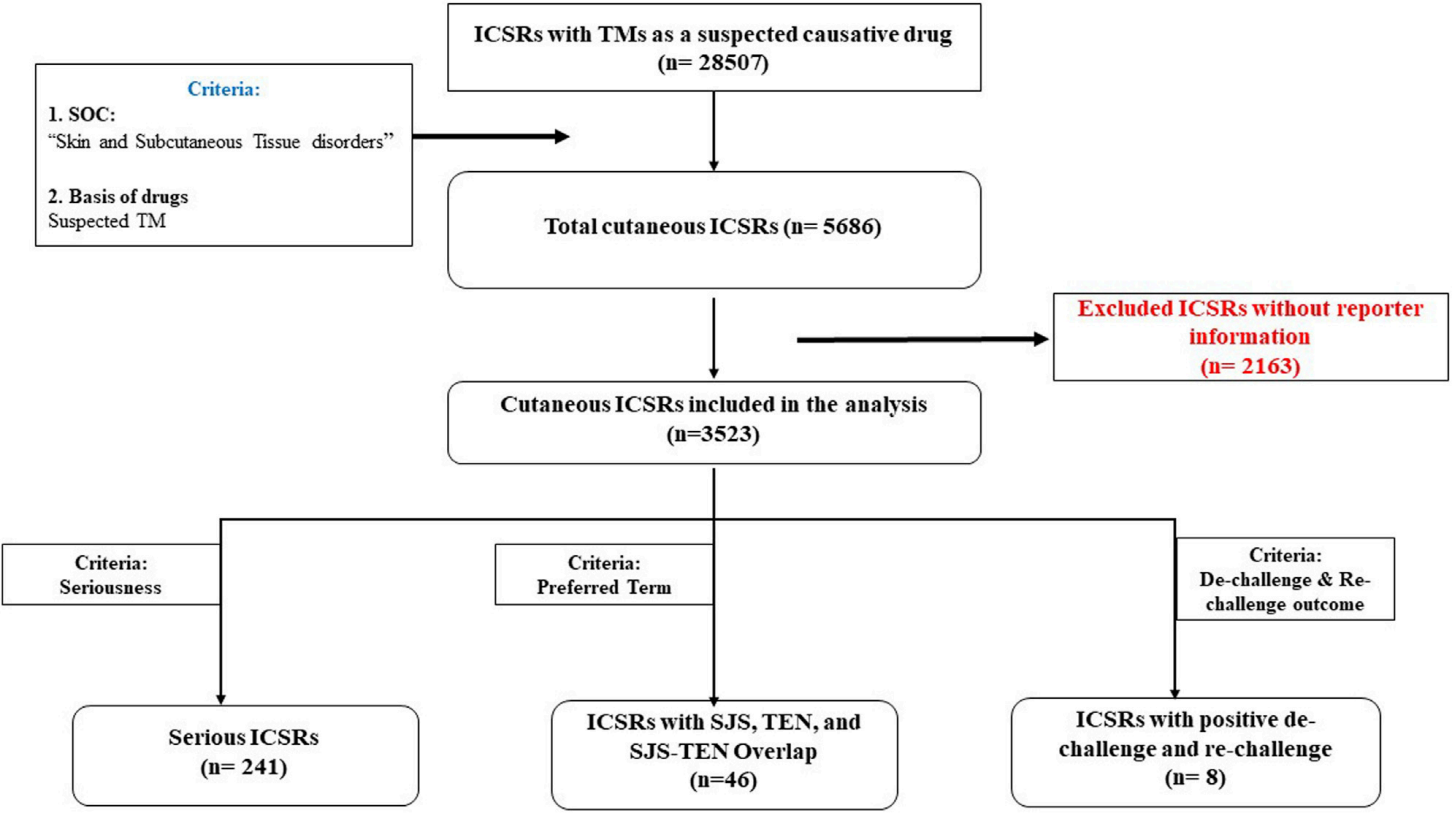
Data flow of suspected cutaneous ADRs with traditional medicines from VigiBase.

The overview of data flow is presented in Figure 1. The age distribution of patients with cutaneous ADRs linked to various TMs is shown in Table 1. A total of 31.4% of the patients were female, and 65.7% were male.

**TABLE 1:** Age distribution of patients with cutaneous ADRs.

Age	Number	Percentage
<18 years	171	4.9
18–44 years	632	17.9
45–64 years	1,205	34.2
≥ 65 years	780	22.1
Not mentioned	735	22.9
Total	3,523	100

A total of 38.8% of suspected cutaneous ADRs were reported by pharmacists, followed by 33.5% reported by physicians or other healthcare professionals and 27.7% from consumers. A lawyer reported one ICSR. A total of 40.2% of patients recovered from ADR. The outcome of ADR was missing in 16.2% ICSRs, and it was mentioned as unknown in 22.6% ICSRs. Death was reported in 5 ICSRs. In 163 (4.6%) cases, ADRs were resolved with sequelae. A total of 437 (7.6%) ADRs were mentioned as serious in 241 (6.8%) patients. The distribution of serious ADRs was higher in females (60.2%) than in males (39%). Serious ADRs were most frequently reported in the age group 45–64 years (36.9%) and 18–44 years (36.9%), followed by ≥ 65 years (13.3%) and <18 years (9.1%).

**FIGURE 2:**
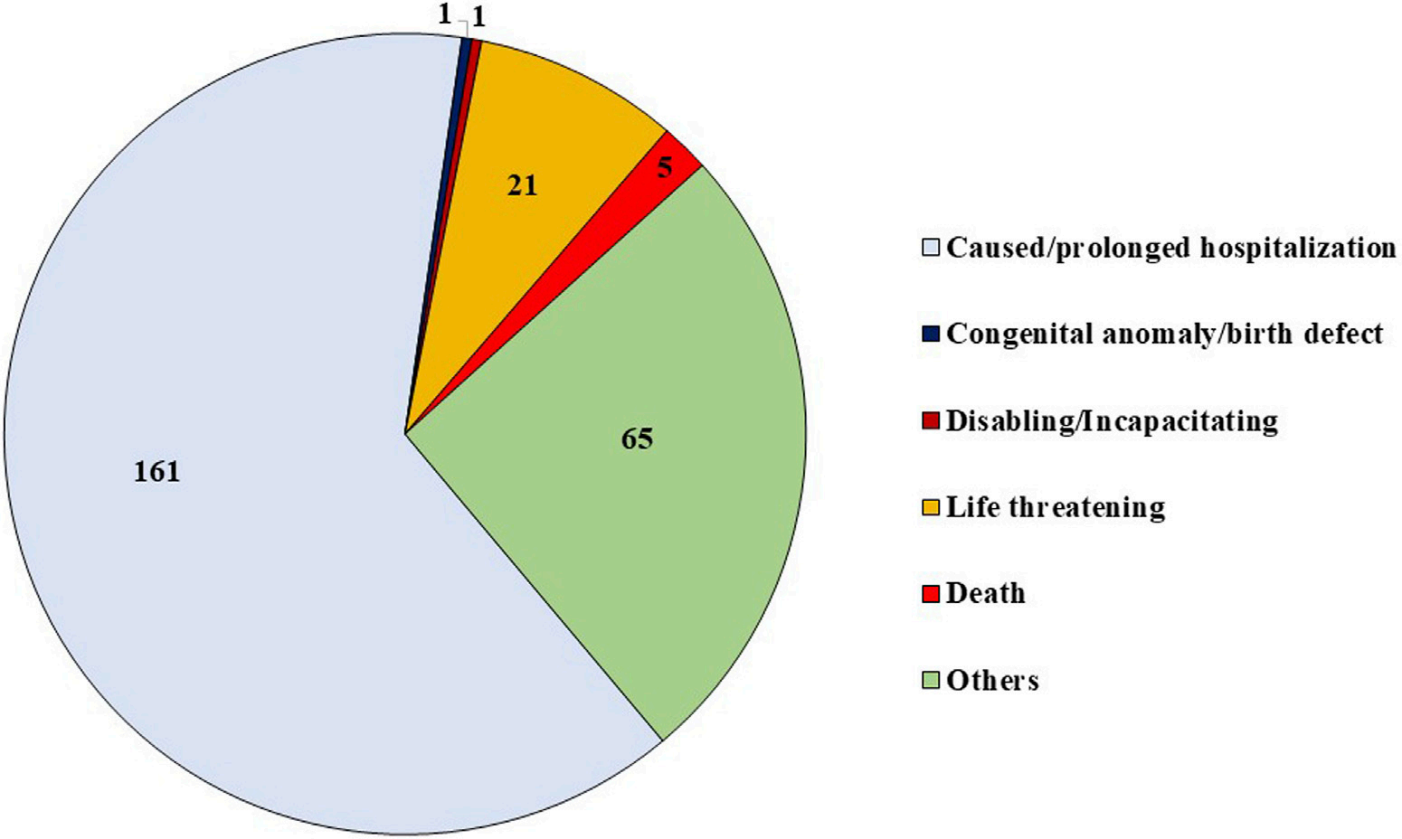
Reported criteria for the seriousness of ADRs.

Multiple criteria for seriousness were reported in 13 out of 241 ICSRs. The distribution of various criteria for considering ADRs as serious is shown in Figure 2. Amongst patients with serious ADRs, 78.4% recovered or were recovering from serious ADRs at the time of reporting, whereas 7.1% were not recovered, 2.1% recovered with sequelae, and 1.2% died of serious ADR. In 9.1% ICSRs, the outcome was unknown.

### Distribution of reported cutaneous ADRs (all vs. serious)

**FIGURE 3:**
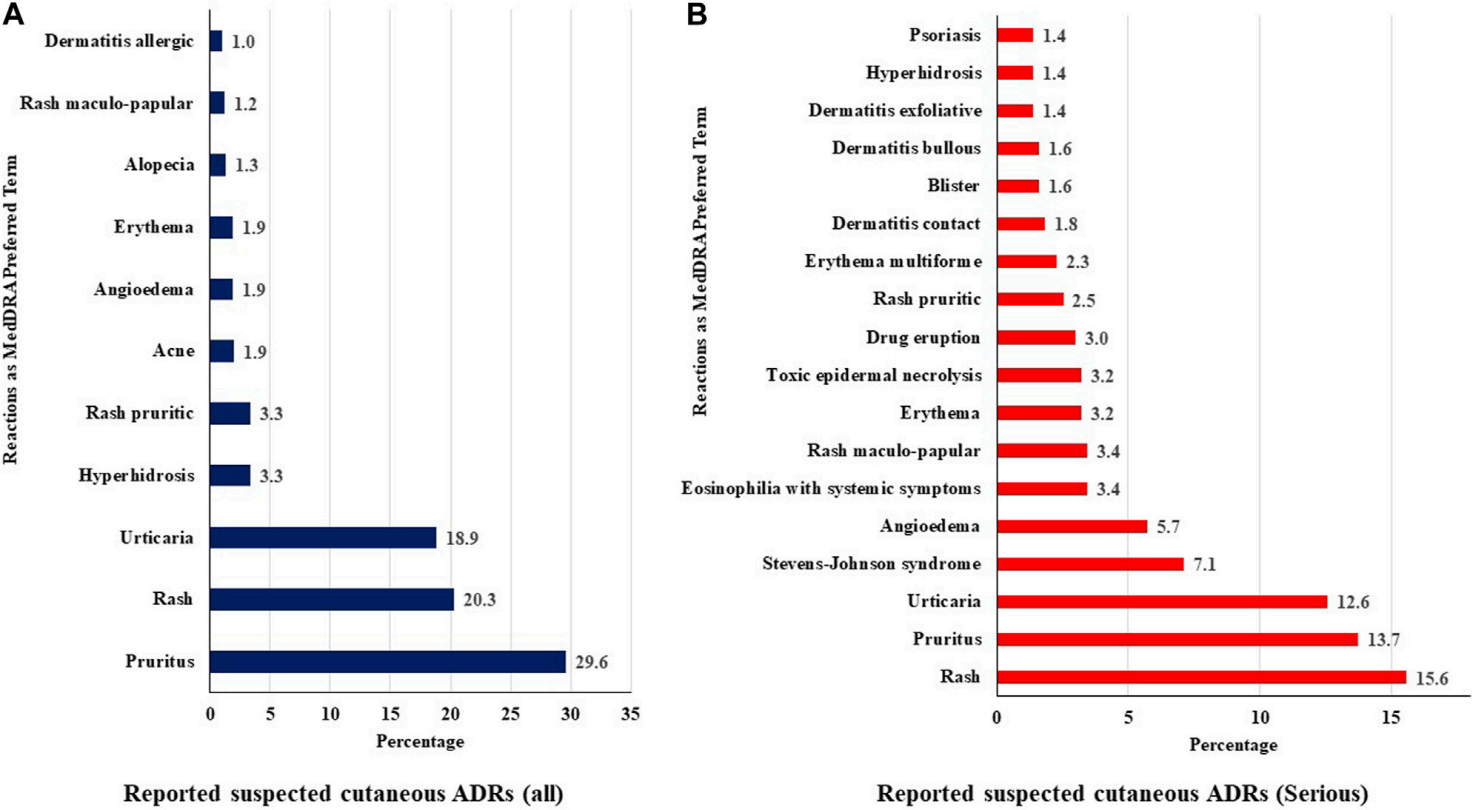
Distribution of suspected cutaneous ADRs (**(A)** all and **(B)** serious) reported in VigiBase.

Based on MedDRA PT, pruritus (29.6%) was the most reported event, followed by a rash (20.3%) and urticaria (18.9%). Angioedema was reported in 1.9% of cases. Figure 3 shows the commonly reported suspected cutaneous ADRs with TMs, including serious ADRs.

### TMs causing cutaneous ADRs

*Artemisia argyi* H.Lév. and Vaniot., was the most suspected botanical drug in reported cutaneous ADRs, with 856 reports, at 14.9%. *Ginkgo biloba* L. (5.1%), Combinations of *Clematis* spp.*+ Prunella vulgaris* L. *+ Trichosanthes kirilowii* Maxim. (4.8%), Combinations of *Corydalis yanhusuo* (Y.H.Chou and Chun C.Hsu) W.T.Wang ex Z.Y.Su and C.Y.Wu. *+ Ipomoea nil* (L.) Roth. (4.1), and *Vitis vinifera* (L.) (4%) were other commonly suspected TMs in reported suspected cutaneous reactions. Non-specified TMs comprised 13.6% of ADRs. The most frequently reported TMs associated with cutaneous ADRs are included in Table 2.

**TABLE 2:** Traditional medicines causing cutaneous ADRs (> 1%).

Suspected medicine (family)	No (%)
**With a single ingredient**	
*Artemisia argyi* H.Lév. and Vaniot. (Asteraceae)	856 (14.9)
*Ginkgo biloba* L. (Ginkgoaceae)	293 (5.1)
*Vitis vinifera* L. (Vitaceae)	228 (4.0)
*Vitex agnus-castus* L. (Lamiaceae)	218 (3.8)
*Silybum marianum* (L.) Gaertn. (Asteraceae)	200 (3.5)
*Viscum album* L. (Santalaceae)	153 (2.7)
*Hedera helix* L. (Araliaceae)	152 (2.6)
*Cimicifuga racemosa* (L.) Nutt; Syn. *Actea racemosa* L. (Ranuunculaceae)	102 (1.8)
*Pelargonium sidoides* DC. (Geraniaceae)	101 (1.8)
*Centella asiatica* (L.) Urb. (Apiaceae)	91 (1.6)
*Angelica* spp. (Apiaceae)	69(1.2)
**With multiple ingredients**	
*Clematis* spp. (Ranunculaceae)*; Prunella vulgaris* L. (Lamiaceae)*; Trichosanthes kirilowii* Maxim. (Cucurbitaceae)	279 (4.8)
*Corydalis yanhusuo* (Y.H.Chou & Chun C.Hsu) W.T.Wang ex Z.Y.Su & C.Y.Wu. (Papaveraceae)*; Ipomoea nil* (L.) Roth. (Convolvulaceae)	238 (4.1)
*Acanthopanax gracilistylus* W.W. Sm. (Syn. *Eleutherococcus nodiflorus* (Dunn.) S.Y.Hu. (Araliaceae)*; Achyranthes bidentata* Blume. (Amaranthaceae)*; Cibotium barometz* (L.) J.Sm. (Cyatheaceae)*; Eucommia ulmoides* Oliv. (Eucommiaceae)*; Glycine* max (L.) Merr. (Fabaceae)*; Saposhnikovia divaricata* (Turcz. ex. Ledeb.) Schischk. (Apiaceae)	218 (3.8)
*Coptis* spp. (Ranunculaceae)*; Hedera helix* L. (Araliaceae)	189 (3.3)
*Achyranthes* spp. (Amaranthaceae)*; Angelica gigas* Nakai. (Apiaceae)*; Carthamus tinctorius* (L.) Asteraceae*; Chaenomeles* spp. (Rosaceae)*; Cinnamomum cassia* (L.) J.Presl. (Lauraceae)*; Clematis* spp. (Ranunculaceae)*; Cnidium officinale* Makino. (Syn. *Ligusticum officinale* (Makino) Kitag.) (Lauraceae)*; Dipsacus asperoides* CY Cheng & Ai. (Dipsacaceae)*; Eleutherococcus senticosus* (Rupr. & Maxim) Maxim. (Araliaceae)*; Gastrodia elata* Blume. (Orchidaceae)*; Gentiana macrophylla* Pall. (Gentianaceae)*; Saposhnikovia divaricata* (Turcz. ex. Ledeb.) Schischk. (Apiaceae)	146 (2.5)
*Glycine* max (L.) Merr. (Fabaceae)*; Persea americana* Mill. (Lauraceae)	102 (1.8)
**Unknown drugs**	
Drug name/s not accepted in who-dd	399 (6.9)
Drug name/s under assessment for who-dd	305 (5.3)
Ayurvedic preparation NOS	79 (1.4)

### ICSRs with positive de-challenge and re-challenge

De-challenge and Re-challenge were reported in 2,226 (63.1%) and 1,146 (32.5%) ICSRs, respectively. In 1,836 (52.1%) reports, reactions abated after the de-challenge of suspected medicine. In 10 (0.3%) ICSRs, the reaction recurred after reintroducing suspected medicines. The suspected medicine-ADR pairs of 8 ICSRs with positive de-challenge and re-challenge are shown in Table 3.

**TABLE 3:** Medicine-ADR pair for which de-challenge and re-challenge both were positive.

Medicine/Botanicals (family)	ADR	No of ICSRs
**Single ingredient-ADR**		
*Aloe vera* (L.) Burm.f. (Asphodelaceae)	Papule	1
	Pruritus	1
*Crocus sativus* L. (Iridaceae)	Urticaria	1
*Hippophae rhamnoides* L. (Elaegnaceae)	Skin lesion	1
*Tinospora cordifolia* (Willd.) Hook.f. & Thomson. (Menispermaceae)	Erythema	1
**Multiple ingredients-ADR**		
*Panax ginseng* C.A. Mey. (Araliaceae); *Zanthoxylum piperitum* (L.) DC. (Rutaceae); *Zingiber officinale* Roscoe. (Zingiberaceae)	Drug eruption	1
**Unknown ingredient-ADR**		
Ayurvedic preparation NOS	Pruritus	1
Herbal anti-acne preparations for topical use	Pruritus	1

### Traditional medicines causing SJS, TEN, and SJS-TEN overlap

SJS, TEN, and SJS-TEN overlaps were reported in 27, 18, and 01 ICSRs, respectively, with TMs. A list of suspected TMs causing SJS and TEN has been presented in Table 4. Only one case of SJS-TEN overlap was reported with the *Rhamnus* plant.

**TABLE 4:** List of suspected TMs for Steven Johnson syndrome (SJS) and Toxic epidermal necrolysis (TEN).

Suspected TMs (family)	SJS	TEN
**Single ingredient**		
*Artemisia argyi* H.Lév. & Vaniot. (Asteraceae)	0	1
*Coix* spp.	-	1
*Commiphora mukul* (Hook. ex. Stocks) Engl. (Burseraceae)	-	1
*Commiphora wightii* (Arn.) Bhandari (Burseraceae)	-	1
*Ginkgo biloba* L. (Ginkgoaceae)	2	-
*Glycine* max (L.) Merr. (Fabaceae)	-	1
*Hedera helix* L. (Araliaceae)	-	1
*Andrographis paniculata* (Burm.f) Nees. (Acanthaceae)	0	1
*Atropa belladonna* L. (Solanaceae)	1	
*Coix lacryma-jobi* L. (Poaceae)	1	-
*Echinacea purpurea* (L.) Moench. (Asteraceae)	1	-
*Phyllanthus amarus* Schumach.& Thonn. (Phyllanthaceae)	1	1
*Saccharomyces cerevisiae*	-	1
*Silybum marianum* (L.) Gaertn. (Astercaeae)	-	1
*Senna alata* (L.) Roxb. (Fabaceae)	1	-
*Valeriana officinalis* L. (Caprifoliaceae)	1	-
**Multiple ingredients**		
*Achyranthes bidentata* Blume. (Amaranthaceae); *Aconitum spp*. (Ranunculaceae); *Alisma orientale* (Sam.) Juz. (Alismataceae); *Cinnamomum cassia* (L.) J.Presl. (Lauraceae); *Cornus officinalis* Siebold & Zucc. (Cornaceae)*; Dioscorea* spp.*; Paeoniax suffruticosa* Andrews. (Paeoniaceae); *Plantago asiatica* L. (Plantaginaceae); *Poria cocos*; *Rehmannia glutinosa* (Gaertn.) DC. (Orobanchaceae)	-	1
*Angelica acutiloba* (Siebold & Zucc.) Kitag. (Apiaceae)*; Atractylodes lancea* (Thunb.) DC. (Asteraceae)*; Bupleurum falcatum* L. (Apiaceae)*; Cnidium officinale* Makino. (Syn. *Ligusticum officinale* (Makino) Kitag.) (Lauraceae)*; Glycyrrhiza* spp. (Fabaceae)*; Poria cocos; Uncaria* spp.	-	1
*Cinnamomum cassia* (L.) J.Presl. (Lauraceae)*; Ephedra* spp.*; Glycyrrhiza* spp. (Fabaceae)*; Paeonia lactiflora* Pall. (Paeoniaceae); *Pueraria lobata* (Willd.) Ohwi. (Fabaceae)*; Zingiber officinale* Roscoe. (Zingiberaceae); *Ziziphus jujuba* Mill. (Rhamnaceae)	1	-
*Aconitum* spp. (Ranunculaceae)*; Atractylodes lancea* (Thunb.) DC. (Asteraceae); *Cinnamomum cassia* (L.) J.Presl. (Lauraceae)*; Glycyrrhiza* spp. (Fabaceae); *Paeonia lactiflora* Pall. (Paeoniaceae)*; Zingiber officinale* Roscoe. (Zingiberaceae); *Ziziphus jujuba* Mill. (Rhamnaceae)	1	-
*Andrographis paniculata* (Burm.f) Nees. (Acanthaceae)*; Angelica dahurica* (Hoffm.) Benth.& Hook.f. ex. Franch.& Sav. (Apiaceae)*; Conioselinum anthriscoides* (H.Boissieu) Pimenov & Kljuykov (Apiaceae); *Ligusticum chuanxiong* SH Qiu., YQ Zeng., KY Pan., YC Tang & JM Xu (Apiaceae)*; Piper longum* L. (Piperaceae)	1	-
*Antelope horn; Arctium lappa* L. (Asteraceae)*; Forsythia* spp. (Oleaceae)*; Glycine* max (L.) Merr. (Fabaceae)*; Glycyrrhiza uralensis* Fisch. ex. DC. (Fabaceae); *Lonicera japonica* Thunb. (Caprifoliaceae)*; Lophatherum gracile* Brongn. (Poaceae); *Mentha canadensis* L. (Lamiaceae)*; Platycodon grandifloras* (Jacq.) A.DC. (Campanulaceae)*; Schizonepeta tenuifolia* Briq. (Lamiaceae)	1	-
*Coptis* spp. (Ranunculaceae)*; Gardenia jasminoides* J.Ellis (Rubiaceae); *Phellodendron* spp. (Rutaceae)*; Scutellaria baicalensis* Georgi (Lamiaceae)	1	-
*Coptis* spp. (Ranunculaceae)*; Hedera helix* L. (Araliaceae)	1	-
*Panax ginseng* C.A. Mey. (Araliaceae)*; Zanthoxylum piperitum* (L.) DC. (Rutaceae); *Zingiber officinale* Roscoe. (Zingiberaceae)	1	1
**Unknown**		
Herbal antivaricose remedies	1	-
Drug name/s under assessment for who-dd	1	3
Ayurvedic preparation NOS	7	2
Unspecified traditional medicine	3	1
Traditional medicine	2	-
Herbal extract nos	1	-

### Indications of herbal medicines

Traditional medicines were prescribed for various conditions. Most medicines were used for multiple health issues in different cases, for example, *Artemisia argyi* H.Lév. and Vaniot. was used for respiratory, gastrointestinal, genitourinary, and musculoskeletal disorders. *Ginkgo biloba* L. was used for central nervous system, cardiovascular, and ear, nose and throat problems. Table 5 enlists the system-wise indications of commonly suspected TMs causing cutaneous ADRs.

**TABLE 5:** Indications for which traditional medicines causing >1% cutaneous ADRs.

Medicines	Reported system-wise indications
**Single ingredient**	
*Artemisia argyi* H.Lév. & Vaniot. (Asteraceae)	Respiratory disorders, Genito urinary disorders, Gynecological problems, CNS and spine disorders, Gastrointestinal disorders, Musculoskeletal problems
*Ginkgo biloba* L. (Ginkgoaceae)	CNS and ENT disorders, ophthalmic disorders, cardiovascular disorders, gastrointestinal disorders
*Vitis vinifera* L. (Vitaceae)	Vascular disorders, musculoskeletal disorders
*Vitex agnus-castus* L. (Lamiaceae)	Gynecological disorders
*Silybum marianum* (L.) Gaertn. (Astercaeae)	Hepatobiliary disorders, CNS disorders, skin disorders, musculoskeletal disorders
*Viscum album* L. (Santalaceae)	Malignancies
*Hedera helix* L. (Araliaceae)	Respiratory diseases
*Cimicifuga racemose* (L.) Nutt. Syn. *Actea recemosa* L. (Ranunculaceae)	Postmenopausal disorders
*Pelargonium sidoides* DC. (Geraniaceae)	Respiratory diseases
*Centella asiatica* (L.) Urb. (Apiaceae)	Skin diseases
*Angelica* spp. (Apiaceae)	Gynecological disorders
**Multiple ingredients**	
*Clematis* spp. (Ranunculaceae)*; Prunella vulgaris* L. (Lamiaceae)*; Trichosanthes kirilowii* Maxim. (Cucurbitaceae)	Musculoskeletal and spine disorders
*Corydalis yanhusuo* (Y.H.Chou & Chun C.Hsu) W.T.Wang ex Z.Y.Su & C.Y.Wu. (Papaveraceae)*; Ipomoea nil* (L.) Roth. (Convolvulaceae)	Burns, cardiovascular diseases, gastrointestinal disorders, musculoskeletal disorders, CNS and spine disorders
*Acanthopanax gracilistylus* W.W. Sm. (Syn. *Eleutherococcus nodiflorus* (Dunn.) S.Y.Hu.) (Araliaceae)*; Achyranthes bidentata* Blume. (Amaranthaceae)*; Cibotium barometz* (L.) J.Sm. (Cyatheaceae)*; Eucommia ulmoides* Oliv. (Eucommiaceae)*; Glycine* max (L.) Merr. (Fabaceae)*; Saposhnikovia divaricata* (Turcz. ex. Ledeb.) Schischk. (Apiaceae)	Musculoskeletal disorders
*Coptis* spp. (Ranunculaceae)*; Hedera helix* L. (Araliaceae)	Acute respiratory diseases
*Achyranthes* spp. (Amaranthaceae)*; Angelica gigas* Nakai. (Apiaceae)*; Carthamus tinctorius* (L.) Asteraceae*; Chaenomeles* spp. (Rosaceae)*; Cinnamomum cassia* (L.) J.Presl. (Lauraceae)*; Clematis* spp. (Ranunculaceae)*; Cnidium officinale* Makino. (Syn. *Ligusticum officinale* (Makino) Kitag.) (Lauraceae)*; Dipsacus asperoides* CY Cheng & Ai. (Dipsacaceae)*; Eleutherococcus senticosus* (Rupr. & Maxim) Maxim. (Araliaceae)*; Gastrodia elata* Blume. (Orchidaceae)*; Gentiana macrophylla* Pall. (Gentianaceae)*; Saposhnikovia divaricata* (Turcz. ex. Ledeb.) Schischk. (Apiaceae)	Musculoskeletal disorders, Neurological disorders
*Glycine* max (L.) Merr. (Fabaceae)*; Persea americana* Mill. (Lauraceae)	Musculoskeletal disorders

## Discussion

This secondary data analysis quantifies and characterizes suspected cutaneous ADRs connected to TMs that were spontaneously reported to the big data repository VigiBase. To the best of our knowledge, the list of TMs that may cause cutaneous ADRs including SJS/TEN is not available in the literature. As TMs are misbelieved to be completely safe, spontaneous reporting of individual events is an important source of getting information on ADRs associated with them (Tabali et al., 2009). The information collected through the spontaneous reporting system in VigiBase helps in generating signals which can be validated later (Chang et al., 2021). The compiled data on suspected cutaneous ADRs with TMs in the present study will provide reference to clinicians and will be helpful to them in linking TMs with cutaneous ADRs during their clinical practice.

Data indicates that TMs could cause a wide range of cutaneous ADRs from pruritus, rash, and urticaria to angioedema, SJS, and TEN. They were reported in VigiBase across all the age groups, with preponderance in the 45–64 years age group. This is may be because of more utilization of TMs by this age group. An earlier retrospective study reported the occurrence of maculopapular exanthema, exfoliative dermatitis, SJS, TEN urticaria, Drug Hypersensitivity Syndrome (DHS), angioedema, and fixed drug eruption amongst patients who received polyherbal formulation (Nair and Varghese, 2019). In an analysis of spontaneous reports from a national pharmacovigilance database of India by Kalaiselvan et al. (2015), skin-related ADRs were commonly reported. The commonly reported suspected cutaneous ADRs were itching, hair loss, dermatitis, rashes, hypopigmentation, nail disorder, lichenoid reaction, and suspected herbal drugs were Mahavat Vidhwansan, Dashmool Kwath/Mahavat Vidhwansan, Aloe, Mustard oil, Garlic, Menthol, Turmeric, Hypericum and unspecified herbal preparation (Kalaiselvan et al., 2015). In a systematic review of ADRs with herbal products reported in randomized controlled trials, reactions like rash, burning skin sensation, and mild irritative contact dermatitis were reported in 6.4% of patients (Lee et al., 2016).

Cutaneous ADRs represent one out of five suspected TMs-connected ADRs. Of all the extracted ICSRs, 19.9% ICSRs were related to suspected cutaneous ADRs in the present analysis. Skin-related ADRs are common and contribute a major quantum of ADRs for both modern medicines and complementary health products (Giardina et al., 2018; Xu et al., 2018). The lists of modern medicines causing various cutaneous ADRs have been available extensively in the literature and regularly updated through various sources of information that can guide clinicians in being careful while prescribing these drugs. However, such information is scarce for TMs. *Artemisia argyi* H.Lév. and Vaniot., *Ginko Biloba* L., *Vitis Vinifera* L. were some commonly reported botanicals to cause skin-related ADRs in this analysis (Table 2).

TMs could cause rare but serious cutaneous ADRs like SJS/TEN. Drugs account for nine out of ten cases of SJS/TEN (Barvaliya et al., 2011; Patel et al., 2013). SJS/TEN are associated with a high incidence of mortality (Kumar et al., 2018). The earlier the causative drug is withdrawn, the better is the prognosis for SJS/TEN (Garcia-Doval et al., 2000). In the current analysis, we have prepared a list of suspected TMs that may cause SJS/TEN. *Artemisia argyi* H.Lév. and Vaniot., *Coix* spp., *Ginko biloba* L., *Commiphora mukul* (Hook. ex. Stocks) Engl., *Commiphora wightii* (Arn.) Bhandari., *Glyccine* max (L.) Merr., *Andrographis paniculata* (Burm.f) Nees., *Phyllanthus amarus* Sch. and Thonn., *Silybum marianum* (L.) Gaertn., *Senna alata* (L.) Roxb., etc., have been reported as suspected medicines in causing SJS/TEN (Table 4). This list will be helpful to clinicians in evaluating the previous drug history while assessing the case of SJS/TEN. In the present study, 46 (1.3%) cases of TM-induced SJS, TEN, and SJS-TEN overlap were reported over a period of 5 years. This suggests the importance of pharmacovigilance activities with TMs. Few case reports have been published where SJS and TEN are reported with Ayurvedic and traditional medicines (Chowdhury et al., 2004; Shivamurthy et al., 2012; Jangra et al., 2020; Oluwo et al., 2020). However, in most cases, the ingredients of medicines were not available. In the case reported by Lim et al. (2018), herbal medication containing deer antlers, ginseng and camphor was found to cause SJS in a 77-year-old male.

Almost 7% of cutaneous suspected ADRs due to TMS were reported as serious in the WHO database. Rash, pruritus, urticaria, SJS, angioedema, and eosinophilia were some commonly reported serious reactions. This suggests being careful while prescribing the TMs to avoid serious consequences. A history of allergic reactions to TMs should be considered to prevent ADRs. The history of consumption of TMs should be considered while evaluating patients with drug-related cutaneous ADRs. In the dataset analyzed, we found 8 ICSRs had positive de-challenge and re-challenge. A definite causal relationship was established for various reactions like erythema, urticaria, pruritus, and drug eruption amongst these ICSRs.

Few reports with unspecified TMs without mentioning ingredients have also been found in the dataset. All efforts should be made to find the ingredients of TMs, as most of the time, herbal formulations contain multiple ingredients. Proper nomenclature is critical for accurately identifying the TMs implicated in ADR reports in a PV database (Wu et al., 2007). In addition to identifying the plant components utilized and the preparation technique in the reports, and afterward, in VigiBase, the use of scientific binomial nomenclature, including botanical authority, is crucial (Farah et al., 2006; Dauncey et al., 2019). However, it is impossible to assign all the information because of several factors: a) the use of common names for TMs in ADR reports submitted by end users and healthcare professionals; b) the preparation made up of crude herbal drugs, and c) not mentioning of the plant species and parts used to make herbal compounds on product labels. Additionally, worries about the effectiveness and safety of these products are brought up by the growing international commerce in TMs (Walker and Applequist, 2012). Raw herbal materials in the supply chain may contain contaminated material, and herbal products may have falsified information (Mishra et al., 2016). The inclusion of plant species not mentioned on the product label in this instance may be the cause of cutaneous ADRs in connection with counterfeit herbal products. Quality control and strict legislation are required. Additionally, it is crucial to create crude drug warehouses to save real botanicals as reference standards (Srirama et al., 2017).

The present study has several limitations. Our study is based on an analysis of spontaneously reported suspected ADRs from VigiBase. This inherently contains limitations of the spontaneous reporting system and retrospective study design. There is a possibility of underreporting. The findings represent the suspicion of the reporter based on the observations made by them. The present analysis does not prove that the suspected TM is directly linked to the particular ADR. It only generates a hypothesis that can be validated in a large-scale cohort study. Our analysis did not include information about the concurrent use of Western medicines or other TMs. Readers should also understand that the source of the data is WHO’s VigiBase but the presented information on suspected cutaneous ADRs with TMs does not represent the opinion of the UMC or the WHO. Regulations of TMs are a major concern, and they also vary from country to country. Authors could not get country-specific data from VigiBase hence, it would not be possible to assess whether regulated or unregulated TMs were causing suspected ADRs. For the safety of TMs, there are multiple other factors that may contribute to the occurrence of ADRs which include misidentification, adulteration, spurious drugs, faulty methods of preparing medicine, etc. No data were available for these aspects so authors could not evaluate them for suspected ADRs.

## Conclusion

In conclusion, TMs may cause a wide range of cutaneous ADRs, including rash, pruritus, urticaria, angioedema, and SJS/TEN, which may have serious consequences also. The present analysis provides an extensive list of TMs, which may cause cutaneous ADRs. Pharmacovigilance activities for TMs should be promoted for better safety monitoring.

## Data Availability

The data analyzed in this study is subject to the following licenses/restrictions: due to UMC’s data protection policy, the datasets used to create this publication are not accessible to the public. The authors are in charge of handling the data in accordance with the UMC’s recommendations for using VigiBase data in studies.
